# A Controllable Plasmonic Resonance in a SiC-Loaded Single-Polarization Single-Mode Photonic Crystal Fiber Enables Its Application as a Compact LWIR Environmental Sensor

**DOI:** 10.3390/ma13183915

**Published:** 2020-09-04

**Authors:** Tianyu Yang, Can Ding, Richard W. Ziolkowski, Y. Jay Guo

**Affiliations:** Global Big Data Technologies Centre (GBDTC), Faculty of Engineering and Information Technology, University of Technology Sydney, Ultimo 2007, Australia; Tianyu.Yang@student.uts.edu.au (T.Y.); Richard.Ziolkowski@uts.edu.au (R.W.Z.); jay.guo@uts.edu.au (Y.J.G.)

**Keywords:** PCF, epsilon-near-zero and epsilon negative, dielectric constant sensing

## Abstract

Near-perfect resonant absorption is attained in a single-polarization single-mode photonic crystal fiber (SPSM PCF) within the long-wave infrared (LWIR) range from 10 to 11 μm. The basic PCF design is a triangular lattice-based cladding of circular air holes and a core region augmented with rectangular slots. A particular set of air holes surrounding the core is partially filled with SiC, which exhibits epsilon near-zero (ENZ) and epsilon negative (ENG) properties within the wavelength range of interest. By tuning the configuration to have the fields of the unwanted fundamental and all higher order modes significantly overlap with the very lossy ENG rings, while the wanted fundamental propagating mode is concentrated in the core, the SPSM outcome is realized. Moreover, a strong plasmonic resonance is attained by adjusting the radii of the resulting cylindrical core-shell structures. The cause of the resonance is carefully investigated and confirmed. The resonance wavelength is shown to finely shift, depending on the relative permittivity of any material introduced into the PCF’s air holes, e.g., by flowing a liquid or gas in them. The potential of this plasmonic-based PCF structure as a very sensitive, short length LWIR spectrometer is demonstrated with an environmental monitoring application.

## 1. Introduction

Among the huge diversity of metamaterials studied in the last 20 years, epsilon-near-zero (ENZ) materials have attracted intense interest, because they are media in which the index of refraction can be engineered to be near zero, i.e., to obtain near-zero-index (NZI) behaviors. Numerous applications utilizing NZI materials have been theoretically predicted and experimentally observed. The interest in artificial media with near-zero parameters was initially triggered by the possibility of developing wavefront shaping and highly directive emitters [1,2,3]. It has since encompassed highly subwavelength tunnelling [4,5]; scattering cancellation (cloaking) [6,7]; wave-matter interactions [8,9]; and, optical [10,11], photonic [12,13], and quantum phenomena [14,15]. Related applications have branched from electromagnetics into other physics areas that are associated with acoustic, mechanical, and thermal wave systems.

While metamaterials have been a popular choice to realize ENZ properties at lower frequencies, e.g., [16], it has also been recognized that there are many materials that naturally exhibit ENZ behavior at higher frequencies [17]. Polaritronic materials, such as transparent conductive oxides (TCO), e.g., ITO and AZO, have been used for applications covering different portions of the near and mid infrared (IR) bands. Polar dielectric and semi-conductor materials, e.g., silicon carbide (SiC), have been employed in the mid and far IR bands. Metals, such as gold and silver, provide opportunities in different portions of the visible range. The strong interest in these ENZ materials at IR frequencies arises from wanting perfect absorption for solar harvesting, biosensing, and thermal imaging. The recognition that simple thin film ENZ coatings could achieve perfect absorption [18,19] has spurred their realizations [20,21,22].

Moreover, ENZ materials have also been used to design polarization maintaining photonic crystal fibers (PCFs) in order to reduce crosstalk in optical fiber communications. For example, a high birefringence PCF has been developed by realizing a high index contrast between its fundamental propagating X-polarized (XP) and Y-polarization (YP) modes through the introduction of ENZ materials into selected air holes [23]. Nevertheless, the high losses that are associated with ENZ materials remain a problem. However, it was found in [24] that they offered an unexpected opportunity in the design of a single-polarization single-mode (SPSM) photonic crystal fiber (PCF). With the ENZ materials being deposited in the cladding region asymmetrically, the fundamental XP, YP, and higher order (HO) modes have distinctly different electric-field (E-field) distributions in the PCF. The geometry of the PCF was optimized to make the YP and HO modes have much larger portions of their fields distributed in the ENZ material than the XP mode. This feature enabled the SPSM PCF outcome, i.e., it made the unwanted YP and HO modes suffer much greater losses than the wanted XP mode. Nonetheless, the lossy ENZ material also introduced noticeable losses to the wanted XP mode and gain material was required to compensate for them, which increased the complexity and reduced the overall practicability of the design.

In this work, we emphasize that the presence of the large losses that are associated with ENZ materials actually can be very advantageous. A passive high-loss plasmonic optical structure based on the THz SPSM PCF design reported in [24] is developed in the long-wavelength infrared (LWIR) regime. SiC is introduced as the ENZ material. An unexpected, extremely strong resonant absorption phenomenon arises in the passive PCF version at a wavelength in the LWIR band on the epsilon-negative (ENG) side of the SiC permittivity’s zero crossing value near 10.3 μm. It is found that the resonance is insensitive to the geometrical parameters, but it is quite sensitive to the relative permittivity variation of the material filling the PCF’s air holes. The reason why this plasmonic resonance appears is explained from a metamaterial-based core-shell perspective and confirmed with simulations. The resulting nearly-perfect absorption, LWIR SPSM PCF has immediate sensor applications, particularly as a very short-length LWIR spectrometer for environmental monitoring. Consequently, its sensitivity performance is demonstrated with water and salt solutions and is compared to state-of-the-art works. This innovative LWIR sensor has comparable performance characteristics, but it requires a much shorter length and exhibits a remarkable tolerance to potential fabrication errors.

## 2. SPSM PCF Configuration and Performance

**Figure 1:**
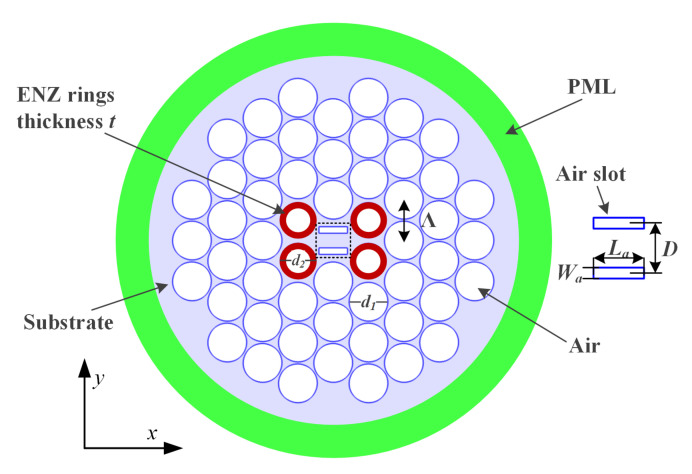
Cross section view of the designed PCF.

The cross section view of the designed SPSM PCF is presented in Figure 1. It is based on a triangular lattice of air holes distributed in the cladding region. SiC, which is represented by the red rings, is deposited into four selected air holes by high-pressure chemical deposition [25] with diameter $d_{2}$ in the first layer of the cladding. These rings have a thickness *t* and an outer diameter $d_{2}$. The remaining air holes share the same diameter $d_{1}$. The lattice constant, Λ, is the distance between the centers of any two adjacent air holes. Two air slots whose dimensions, $L_{a}\times W_{a}$, are set centrally in the core region. The distance between the centers of the two slots is *D*.

All of the simulations were performed with the COMSOL Multiphysics simulator. The PCF configuration was built with the two-dimensional (2D) modeling function in the Radio Frequency module of COMSOL. It is surrounded by a perfect matched layer (PML) whose thickness is 15% of the radius of the cladding and which acts as the absorbing boundary condition region for COMSOL’s finite element solution algorithm. It is highlighted in green in the figure. The Eigenmode Solver was adopted to obtain the propagation constants of the guided modes. The maximum mode number was set to 20 to save time once we learned how many modes actually played a significant role in the calculations, which was generally only around 5 or 6. The target solution resolution was set at $10^{-6}$ and was reached generally in four iterations. The software was then used to obtain the field distributions. The internal post-processing tools provided a means to obtain the total loss values. We provided the internal postprocessing software the appropriate expressions to calculate the contributions to it from the confinement loss (CL) and the effective material absorption loss (EML). The design parameters are all defined relative to the lattice constant. Their optimized values are: Λ = 8.3 μm, $d_{1}$= 0.95 Λ, $d_{2}$= 0.86 Λ, $L_{a}$= 0.76 Λ, $W_{a}$ = 0.2 Λ, *D* = 0.56 Λ, and *t*= 0.13 Λ. The radius of the fiber is 4.6 Λ and the thickness of the PML is 0.7 Λ, i.e., ∼15% of that radius.

**Figure 2:**
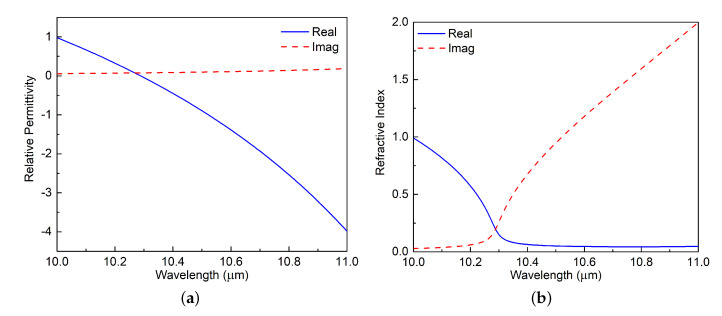
(**a**) The relative permittivity and (**b**) refractive index of SiC.

The polaritonic equation describes the relative permittivity of SiC [26]:

(1) $$\begin{array}{c} \varepsilon \left(\omega \right)=\varepsilon_{\infty }\;\frac{\omega^{2}-\omega_{LO}^{2}+i\gamma \omega }{\omega^{2}-\omega_{TO}^{2}+i\gamma \omega } \end{array}$$

where the longitudinal optical phonon frequency $\omega_{LO}$ = 972 cm^− 1^, the transverse optical phonon frequency $\omega_{TO}$ = 796 cm^− 1^, the damping coefficient γ = 3.75 cm^− 1^, and the infinite-frequency permittivity $\varepsilon_{\infty }$ = 6.5. Figure 2a shows its real and imaginary parts of the relative permittivity and Figure 2b shows the corresponded real and imaginary parts of the refractive index; they both illustrate the dispersive behavior of SiC. The zero crossover point occurs near 10.3 μm. For longer wavelengths, the real part of ε decreases to negative values gradually and, thus, the SiC becomes an ENG substrate with near-zero-index (NZI) attributes that supports plasmonic effects. On the other hand, the imaginary part of ε is large in the range from 10 to 11 μm, i.e., it is on the order of $10^{-1}$. Consequently, the material loss of the SiC in this band is large. To match the SPSM PCF design to these wavelengths, a chalcogenide glass was selected as the substrate [27]. It has a real part permittivity around 6.76 in the LWIR wavelength range of interest. Its material loss is negligible, especially in comparison to that of SiC.

**Figure 3:**
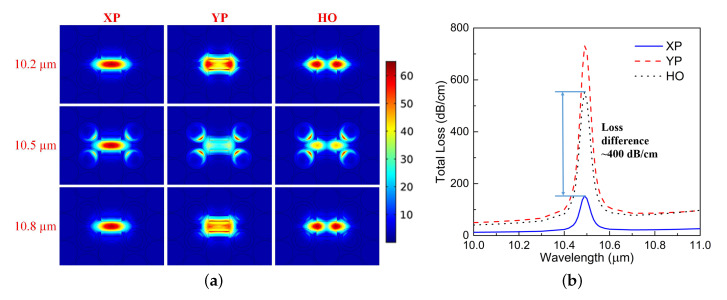
(**a**) E-field distribution of three of the single-polarization single-mode photonic crystal fiber (SPSM PCF) mode types at different wavelengths very near to and on both sides of its resonance frequency. (**b**) Loss properties of all three mode types.

The PCF design in Figure 1 supports two fundamental propagating modes, i.e., the XP and YP modes. However, it also embraces a number of HO modes, which appear in different portions of the operational band. Only the ones whose loss values are low enough to affect the outcomes are considered in the following. The E-field distribution of its XP, YP, and HO modes at 10.2 μm, 10.5 μm, and 10.8 μm are shown in Figure 3a.

One first observes that the E-field distribution of the three modes is distinctly different. This difference is a consequence of the asymmetry of the material distribution in the cladding [24]. Furthermore, the proper arrangement and size of the air slots in the core region further enhances this difference. Consequently, a large loss difference between the wanted XP mode and other unwanted modes is obtained with the optimized structure. One then observes from the field distributions that the largest difference between the wanted XP mode and the other mode types occurs at 10.5 μm. In particular, the YP and HO modes have a larger portion of their fields distributed in those SiC rings. Because the SiC is very lossy within the LWIR wavelength range of interest, the YP and HO modes will have much higher losses than the XP mode at 10.5 μm.

The total loss (TL) of the XP, YP, and HO modes are shown in Figure 3b. These TL values represent the sum of the CL and EML contributions, as explained in [24]. It is observed that there is a general loss difference between the wanted and unwanted modes of more than 28 dB/cm over the entire band, which guarantees the SPSM behavior. Moreover, an unexpected large resonant absorption response is attained at 10.49 μm for all three mode types. In a narrow band around the resonance wavelength, the loss difference is as high as ∼400 dB/cm.

## 3. Resonance Phenomena Analysis

Extensive parameter sweeps of the geometrical dimensions have been carried out to investigate what causes the resonance and to study the factors that influence its wavelength. It is found that variations of the dimensions of the PCF design itself generally have little effect on the resonance wavelength. On the other hand, any material loading of the PCF’s air hole regions has a significant impact on it.

### 3.1. Parameter Sweep of Geometrical Parameters

**Figure 4:**
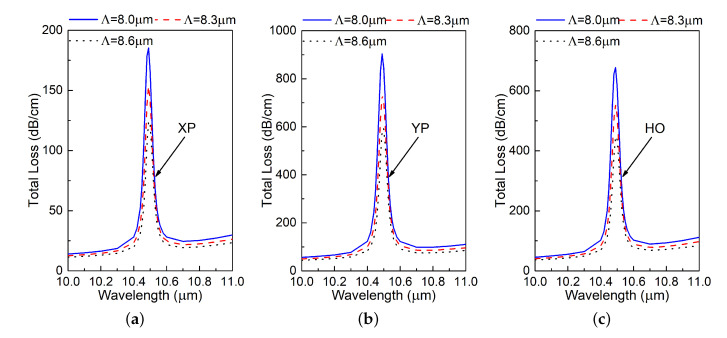
Simulated total loss (TL) values of the (**a**) X-polarized (XP), (**b**) Y-polarized (YP), and (**c**) higher order (HO) modes of the designed PCF as functions of the source wavelength for different values of the lattice constant Λ.

Figure 4 shows the total loss over the wavelength band of interest for different values of Λ. Changing Λ scales the entire PCF to be larger or small as the design parameters are all defined relative to Λ. According to the “PCF scaling principle” detailed in [28], scaling the dimensions of a PCF will shift its working wavelength without changing its performance characteristics, e.g., its birefringence, effective material loss, and confinement loss. However, according to Figure 4a, b, and c, it is clear that the resonance wavelength of all the modes essentially remains the same. This indicates that the absorption resonance is not an intrinsic property of the PCF.

**Figure 5:**
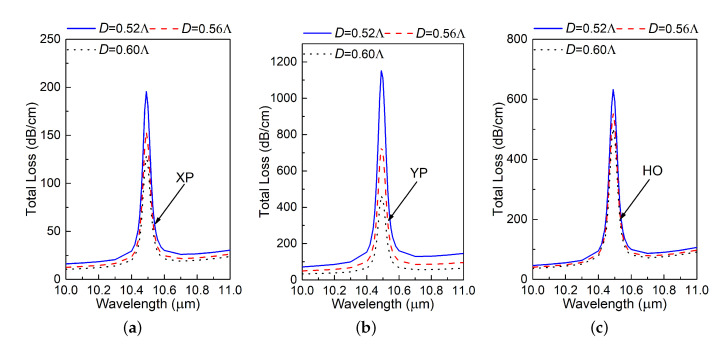
Simulated TL values of the (**a**) XP, (**b**) YP, and (**c**) HO modes of the designed PCF as functions of the source wavelength for different distances *D* between the two air slots.

Furthermore, changing one dimension with all other dimensions remaining the same does not change the resonance wavelength either. For example, Figure 5 shows the simulation results of the TL for XP, YP and HO modes when *D* is varied. Nevertheless, different *D* values result in different magnitudes of the resonance. Parameter studies of all the other design dimensions, except the thickness of the SiC rings (*t*), yielded the same outcomes. Small changes in the design’s dimensions basically have little impact on the absorption resonance wavelength. Note that the associated variations in the TL magnitudes have little practical importance given their very large values. This is particularly important for tolerances that are associated with any eventual fabrication of the design.

**Figure 6:**
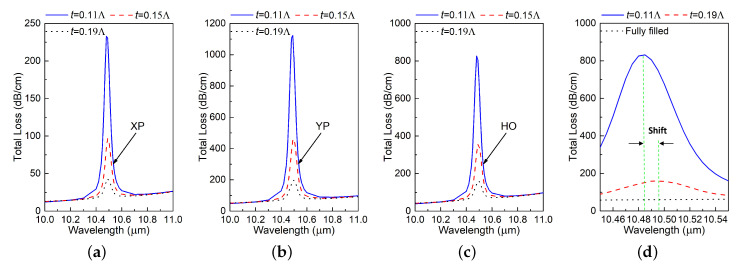
Simulated TL values of the (**a**) XP, (**b**) YP, and (**c**) HO modes of the designed PCF as functions of the source wavelength for different thicknesses of the SiC rings, *t*. (**d**) Zoom-in of the TL values for the HO mode in (**c**) from 10.45 to 10.55 μm.

It was determined that the ring thickness *t* does have an effect on the resonance wavelength, but that it is minor. The corresponding parameter studies are summarized in Figure 6, particularly in the zoom-in plot given in Figure 6d. As *t* increases, the resonance wavelength red-shifts and the magnitude of the loss becomes smaller. Notably, the resonance completely disappears as the holes are filled completely with SiC.

### 3.2. Parameter Sweep of the Relative Permittivity of the Medium in the Enz-Loaded Holes

**Figure 7:**
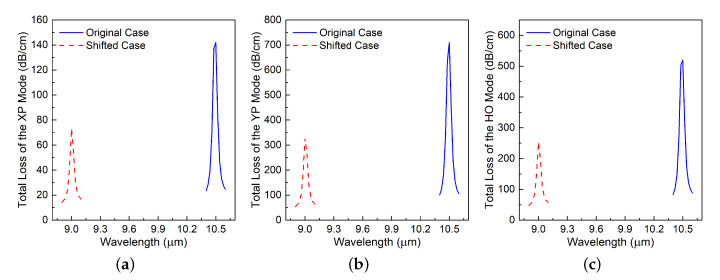
Simulated TL values of the (**a**) XP, (**b**) YP, and (**c**) HO modes in the long-wavelength infrared (LWIR) range. The plasmonic resonance behavior, which is centered at 10.4 μm in the original SiC case (blue), is maintained when the material dispersion of the epsilon negative (ENG) rings is shifted to a smaller wavelength LWIR band centered at 9.0 μm.

It was initially assumed that the resonance was caused by the material characteristics of the ENZ material. To investigate this assumption, the SiC dispersion curves that are shown in Figure 2 were shifted to a smaller wavelength LWIR band, 8.9 to 9.1 μm, from 10.4 to 10.6 μm. Figure 7a–c present the outcome for XP, YP, and HO modes, respectively. It is observed that the resonance wavelength also shifted for the XP, YP, and HO modes. While the peak absorption level at the resonance wavelength, 9.0 μm, is smaller than the original value, it nonetheless remained quite large. This result implies that the material characteristics of the ENZ rings affect the resonance behavior.

**Figure 8:**
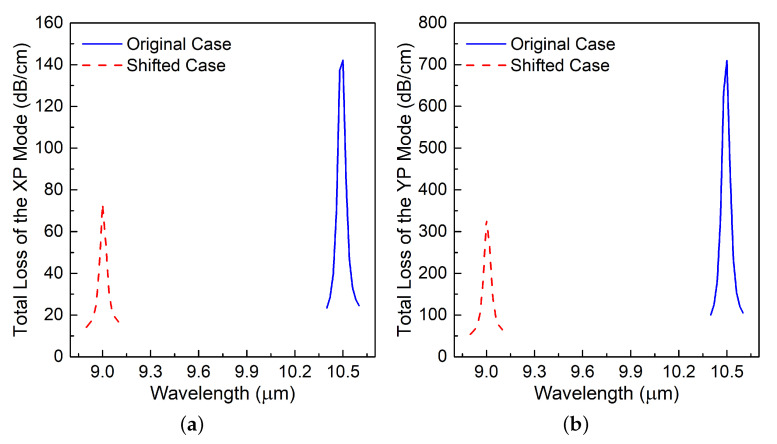
Resonance behavior of the XP mode in the LWIR range when the relative permittivity of the medium in the ENG-loaded rings is changed. (**a**) Re$\left(\varepsilon_{1}\right)$ is changed with Im$\left(\varepsilon_{1}\right)$ = 0. (**b**) Im$\left(\varepsilon_{1}\right)$ is changed with Re$\left(\varepsilon_{1}\right)$ = 1.1.

Consequently, it was then investigated how the resonance is changed when the air in the SiC-loaded holes is changed to a medium with different material characteristics. Parameter sweeps of the real and imaginary parts of the relative permittivity of the medium in the SiC-loaded holes were conducted. The resonance wavelength shift of the XP mode for five different Re$\left(\varepsilon_{1}\right)$ values with Im$\left(\varepsilon_{1}\right)$ = 0 is illustrated in Figure 8a. It is observed that the resonance wavelength is very sensitive to the Re$\left(\varepsilon_{1}\right)$ values of the medium. Figure 8b illustrates the change in the resonance wavelength for different Im$\left(\varepsilon_{1}\right)$ values when the core relative permittivity is fixed at Re$\left(\varepsilon_{1}\right)$ = 1.1. The simulation results indicate that a variation in Im$\left(\varepsilon_{1}\right)$ only slightly changes the resonance wavelength until this loss factor becomes quite large. On the other hand, the TL peak value decreases as Im$\left(\varepsilon_{1}\right)$ increases. Basically, a larger loss factor significantly detunes the resonance, which is a typical behavior that is associated with any normal resonance. It was found that these features are shared for the XP, YP, and HO modes. Thus, the results of the YP and HO modes are not explicitly presented.

### 3.3. The Resonant Cylindrical Core-Shell Theory

The conclusion of the parameter studies is that the resonance behavior is not an intrinsic property of the PCF, but is mainly determined by the four SiC-loaded air holes. In fact, a PCF without these SiC-loaded holes will not exhibit the same resonance behavior. These observations motivated us to investigate the SiC-loaded holes as an independent structure.

**Figure 9:**
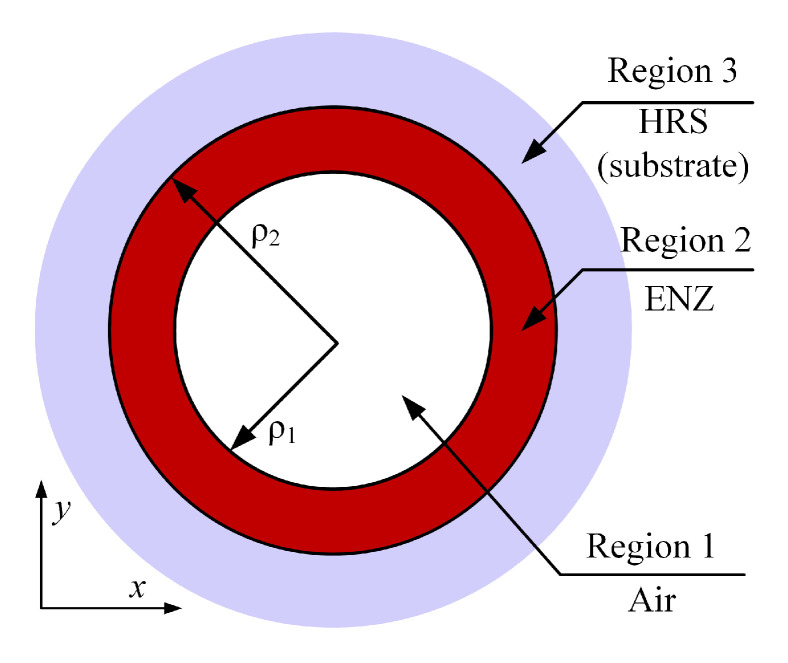
Cylindrical core-shell scattering geometry corresponding to the PCF structure.

The basic geometry of each SiC-loaded air hole is essentially a cylindrical core-shell configuration where Region 1 is air, Region 2 is the SiC, and Region 3 is the chalcogenide glass, as shown in Figure 9. Referring to the resonant scattering and radiating cylindrical core-shell configurations studied previously [29,30], a plasmonic resonance is obtained for the *m*-th multipole mode of such a core-shell geometry when the ratio of the radii $\rho_{1}$ and $\rho_{2}$ satisfies the relation:

(2) $$\begin{array}{c} \frac{\rho_{1}}{\rho_{2}}=\sqrt[{2m}]{\frac{\left(\varepsilon_{2}+\varepsilon_{1}\right)\left(\varepsilon_{2}+\varepsilon_{3}\right)}{\left(\varepsilon_{2}-\varepsilon_{1}\right)\left(\varepsilon_{2}-\varepsilon_{3}\right)}}. \end{array}$$

For the SiC-loaded holes and the wavelengths of interest in this work, the material parameters of the core and exterior regions of the corresponding core-shell configuration: $\varepsilon_{1}$ = 1.0 and $\varepsilon_{3}$ = 6.76, are independent of the wavelength. The ratio of the corresponding radii is $\rho_{1}/\rho_{2}$ = 0.697. Because the SiC is dispersive, $\varepsilon_{2}$ changes with the wavelength according to Figure 2. Given the fixed values of $\varepsilon_{1}$, $\varepsilon_{3}$, and $\rho_{1}/\rho_{2}$, there must be specific values of $\varepsilon_{2}$ that will satisfy Equation (2). We set

(3) $$\begin{array}{c} {\left(\frac{\rho_{1}}{\rho_{2}}\right)}^{2m}=A, \end{array}$$

Equation (2) can then be rewritten as

(4) $$\begin{array}{c} \left(A-1\right){\varepsilon_{2}}^{2}-\left(A+1\right)\left(\varepsilon_{1}+\varepsilon_{3}\right)\varepsilon_{2}+\left(A-1\right)\varepsilon_{1}\varepsilon_{3}=0. \end{array}$$

This is a quadratic equation with one unknown, $\varepsilon_{2}$. There are two standard real solutions of this equation:

(5) $$\begin{array}{c} \varepsilon_{2}=\frac{-b\pm \sqrt{b^{2}-4ac}}{2a}, \end{array}$$

where

(6) $$a=A-1,\;b=-\left(A+1\right)\left(\varepsilon_{1}+\varepsilon_{3}\right),\;c=\left(A-1\right)\varepsilon_{1}\varepsilon_{3}.$$

Because the values of $\varepsilon_{1}$, $\varepsilon_{3}$, and $\rho_{1}/\rho_{2}$ are all known, the specific values of $\varepsilon_{2}$ of the two solutions can be obtained for different *m*. Note that both of the $\varepsilon_{2}$ solutions will always be negative values given the conditions that $\rho_{1}/\rho_{2}<1$ and $\varepsilon_{1}<\varepsilon_{3}$. This feature indicates that an ENG material is required in order to satisfy the resonance condition. Once the $\varepsilon_{2}$ values associated with the resonance of the *m*-th multipole mode are determined, one can simply substitute them into Equation (2) and use it and Figure 2 to determine the corresponding resonance wavelengths.

The two calculated $\varepsilon_{2}$ solutions for several modes and the corresponding resonance wavelengths are summarized in Table 1. Because there are two negative values of $\varepsilon_{2}$ for any *m* value, the same resonance mode occurs at the two indicated wavelengths. Notice that all of the Solution 1 wavelengths fall inside the frequency range of interest while all of the Solution 2 ones are outside of it at longer wavelengths. Additionally, note that the imaginary parts of $\varepsilon_{1}$, $\varepsilon_{2}$, and $\varepsilon_{3}$ were assumed to be zero in these calculations. Because the actual values of the imaginary part of the permittivity are much smaller than the real parts for the wavelengths of interest, the discrepancy from considering them or not is quite small.

We emphasize that the *m*-th order modes of the core-shell model are not the same as the PCF modes. Recall that the peak of the resonance in Figure 3 is at 10.49 Resonance Mode Number for the XP, YP and all HO modes of the PCF. Now referring to Table 1 it is clear that the m=4-th mode of the core-shell structure dominates this resonance behavior. The resonance is broad because there are several neighboring modes of the SiC-loaded core-shell structure that also contribute to the absorption behavior.

### 3.4. Comparisons between the Simulation and Theoretical Results

The parameter sweep results obtained by simulating the performance with the COMSOL Multiphysics simulator are compared with the theoretical results to further validate the resonant core-shell theory. Figure 6 shows the changes of the resonance wavelength as the thickness of the SiC rings, *t*, varies. We know from Equation (2) and Figure 9 that changing *t* actually changes the ratio $\rho_{1}/\rho_{2}$. This variation, in turn, leads to a different $\varepsilon_{2}$ value that satisfies the resonance condition along with a shift in the resonance wavelength.

**Figure 10:**
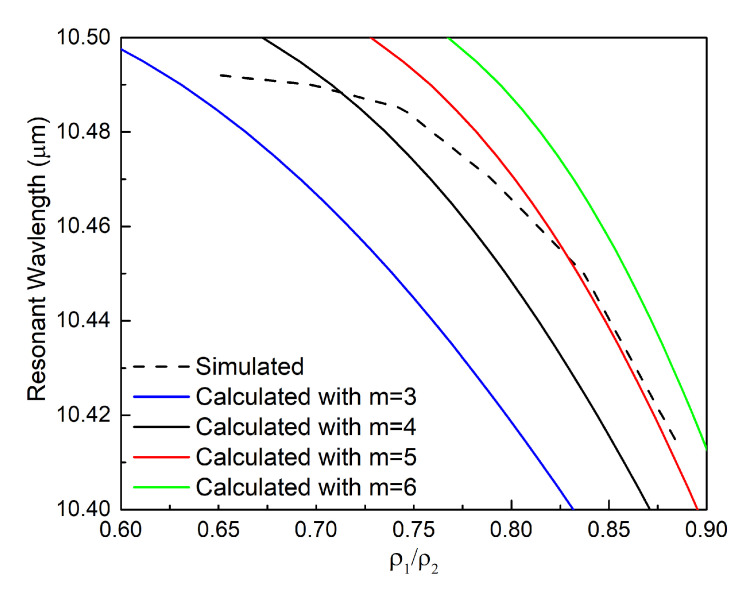
Calculated and simulated variations of the absorption resonance wavelength with the ratio $\rho_{1}/\rho_{2}$ for different core-shell mode numbers *m*. The dashed line represents the COMSOL simulation results; the solid lines represent the analytical results.

The resonance wavelengths were calculated for different $\rho_{1}/\rho_{2}$ and *m* values to understand which core-shell mode dominates the resonant absorption. These results are plotted in Figure 10 and compared with the simulated results. It is observed that the simulated curves share similar trends with the theoretically calculated ones. The simulated wavelengths at the resonance peak generally agree with the calculated results with either *m* = 4 or 5, i.e., the resonance is dominated by either the 4th or 5th resonance mode for different values of $\rho_{1}/\rho_{2}$.

## 4. Sensing Application

Infrared (IR) absorption spectroscopy as a physical sensing approach is an established precise and reliable quantitative analysis technique in order to detect substances in multi-component gas and liquid mixtures. Notable application areas of current importance include environmental and security monitoring, e.g., to detect the presence of toxic components in water and determine the air quality in cities and buildings. It is also useful for process control and quality assurance in the chemical, pharmaceutical, hospital, and food industries. With growing concerns about the quality of local water supplies for drinking and agriculture, miniaturized absorption spectrometers are highly desired for compact applications, e.g., lab-on-chip, and they require significantly reduced light-matter interaction lengths [31].

As illustrated in Figure 8, the wavelength at which the maximum resonant absorption occurs has been found to be very sensitive to the relative permittivity of the medium filling the SiC-loaded holes, i.e., Region 1 in Figure 9. These results provided the stimulus to consider the develop LWIR SPSM PCF as a LWIR spectrometer for liquid and gas monitoring applications by replacing the air in the SiC-loaded holes with a liquid or a gas. In the neighborhood of 10.6 μm, CO_2_ lasers and quantum cascade lasers (QCLs) are ideal light sources for such a LWIR spectrometer application. The CO_2_ laser systems are tunable and can be extremely powerful [32,33]. On the other hand, QCLs have generally replaced CO_2_ lasers for low power applications in that spectral range [34,35]. Moreover, high power QCLs are also available [36,37]. Given the more compact nature of QCL systems, they would be the preferred choice for the intended LWIR compact spectrometer. We have elected to consider a 5.0 mm long PCF for the spectrometer design when considering the fact that a longer PCF introduces more losses and a too short PCF would be difficult to fabricate. Because the TL of the YP and HO modes are much larger than that of the XP mode, the XP mode is the only one left at the outcome. Consequently, the XP mode shift would be the easiest one to be detected in a LWIR spectrometer application.

To investigate its sensing performance, five liquids with different relative permittivity values were selected to fill the air holes in the PCF design. It was found that there was little difference in the simulated shift of the resonant peak whether only the SiC-loaded holes were filled with fluid or all of the holes were. Therefore, because filling all of the air holes in the PCF would be more convenient for a practical realization, the design results were obtained for the PCF with all of its air holes filled with the fluid under test. The test liquids were pure water [38] as the reference case, heavy water [39], and aqueous solutions having 1M, 3M, and 5M salt (NaCl) [40] concentrations.

**Figure 11:**
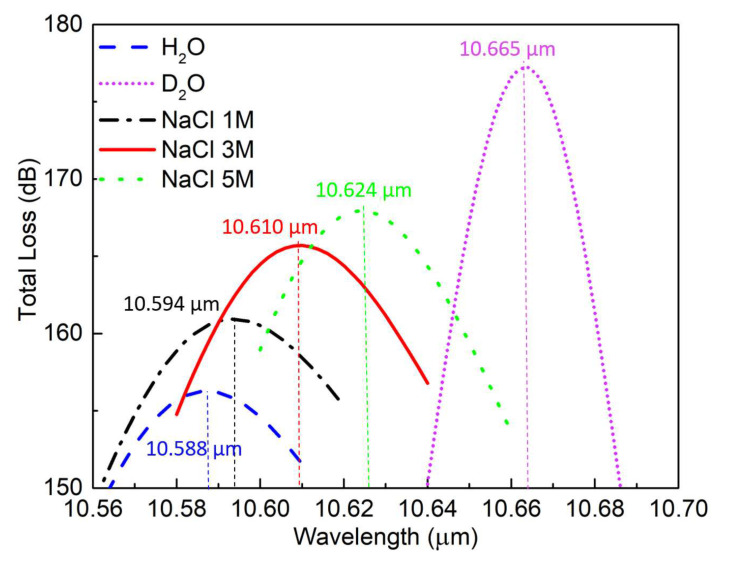
Resonance peak shift when the air in the PCF holes is replaced with the indicated five different liquids. The absorption peak wavelengths are identified.

Figure 11 shows the consequent absorption peaks of the XP mode for these five solutions The dispersion properties of these water-based materials were taken into account; the data were obtained at 22 °C and standard atmospheric pressure. Clear shifts in the peak position are easily distinguished. They are red-shifted, respectively, 6, 22, 36, and 77 nm relative to the peak wavelength of the pure $H_{2}O$.

**Figure 12:**
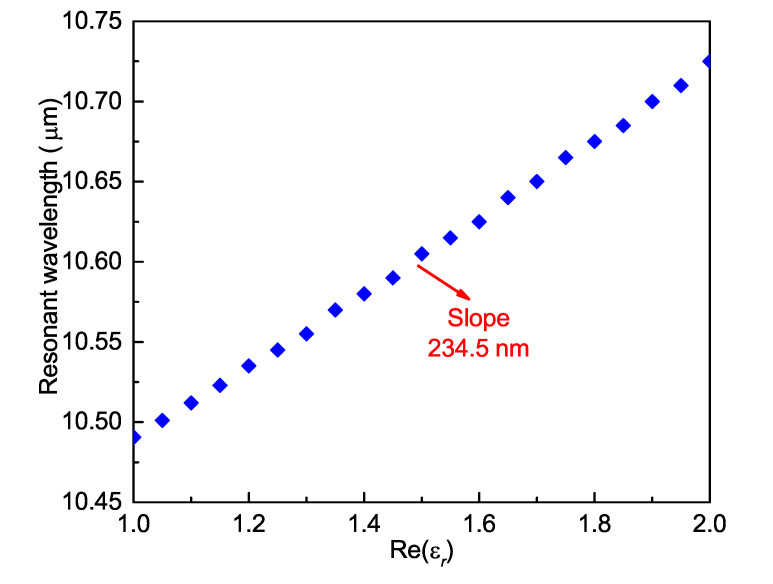
Sensitivity of the SiC-loaded SPSM PCF spectrometer. The slope of the resonance peak wavelength versus the Re($\varepsilon_{r}$) determines its relative permittivity (RPU) value.

The wavelength sensitivity of a sensor is the wavelength shift per unit variation of the relative permittivity (RPU) of the materials being considered. The real part of permittivity of the medium filling in the air holes of the SiC-loaded SPSM PCF was varied from 1.0 to 2.0, which corresponds to a refractive index range from 1.0 to 1.41, with its imaginary part being set to zero to determine its wavelength sensitivity. Figure 12 presents the consequent variation in the resonance wavelength of the absorption peak. The wavelength variation is clearly linear with respect to Re$\left(\varepsilon_{r}\right)$. The sensitivity, i.e., the slope, is 234.5 nm/RPU. However, it is more conventional to express the sensitivity in terms of refractive index units (RIUs). The corresponding value is 566.4 nm/RIU. Another important performance index of the sensor is the sensitivity per linewidth [41]. The linewidth is defined as the full-width at half-maximum, i.e., the wavelengths at which the absorption level is 3 dB lower than the peak value of the resonance. The calculated linewidth is 16 nm when a 5.0 mm long PCF is used for the spectrometer. The corresponding sensitivity per linewidth is 35.4.

The proposed ENG-loaded SPSM PCF spectrometer was compared with comparable state-of-the-art sensors based on measuring the refractive index change of the material being sensed. Table 2 summarizes these comparisons; they show that the sensitivity value of the proposed spectrometer is better than those reported in [42,43,44,45,46,47] and it is comparable to the highest ones in [48,49,50]. Its sensitivity per linewidth value is average in comparison. On the other hand, the detection range is superior, the lowest index value having been demonstrated in Figure 8a. Furthermore, the proposed spectrometer has another two advantages. First, the absorption resonance performance is not sensitive to small changes in the dimensions of the structure, i.e., it would have a high tolerance to any fabrication errors. Second, the SPSM behavior of the PCF naturally eliminates measurement inaccuracies that are introduced by higher order modes, an issue not even considered for the comparison designs.

## 5. Conclusions

In this work, a SPSM PCF at the LWIR wavelengths was developed. The innovation of the design is the loading of selected air holes in the first ring of the cladding of the PCF with SiC rings. These rings exhibit ENZ and ENG properties in the LWIR wavelength region of interest centered at 10.5 μm. They produced an extremely large plasmonic resonance that facilitates near-perfect absorption at those LWIR wavelengths. The parameter studies that were conducted to optimize the design and investigate the cause of the resonance were detailed. It was demonstrated that each ENG-ring filled air hole formed a resonant plasmonic core-shell structure in the glass background. It was shown that, while the resonance wavelength is quite stable with the changes in the PCF’s physical dimensions, it is strongly affected by the material dispersion of the SiC and the permittivity of any medium introduced into the SiC-loaded air holes, as well as all of the other air holes in the cladding. It was also shown that the closely spaced modes of the plasmonic core-shell structures produced the large width of the absorption resonance. The core-shell model’s analytical predictions were confirmed with the simulated results of the entire SPSM PCF structure. Because of the demonstrated sensitvity of the plasmonic-based absorption resonance to the relative permittivity of the medium loading the air holes of the PCF and its high tolerance to potential fabrication errors, a LWIR sensor application was identified and explored. It was illustrated with salt-impregnated water solutions that the ENG-loaded SPSM PCF spectrometer would be a highly sensitive, compact environmental monitoring sensor. In contrast to other comparable devices, remarkable performance characteristics were achieved with this compact LWIR spectrometer for the reported liquid sensor, environmental monitoring application. Nevertheless, its performance with gases at LWIR wavelengths only achieved an average sensitivity. A review of the literature suggests that transitioning the design to mid-IR wavelengths where a number of materials with similar ENG-ENZ properties exist, such as the noted TCOs, and where accurate gas sensors have been reported may be a fruitful next research direction. We hope to report a related SPSM PCF design in the mid-IR with even higher sensitivity for environmentally important gas detection applications in the near future.

## Figures and Tables

**Table 1:** Calculated values of $\varepsilon_{2}$ that satisfy the resonance condition, Equation (2), for the SiC-loaded core-shell structure for different mode numbers *m* and the corresponding resonance wavelengths.

Resonance Mode Number (*m*)		1	2	3	4	5	6
Solution 1	$\varepsilon_{2}$	−0.31	−0.56	−0.75	−0.87	−0.93	−0.97
	$\lambda_{\mathrm{res}}$ (μm)	10.37	10.43	10.47	10.49	10.512	10.516
Solution 2	$\varepsilon_{2}$	−22.17	−12.01	−9.03	−7.82	−7.26	−7.00
	$\lambda_{\mathrm{res}}$ (μm)	11.30	11.32	11.36	11.44	11.60	11.92

**Table 2:** Comparisons Between the Resonant ENG-loaded SPSM PCF Sensor and Comparable State-of-the-Art Devices.

0]* Reference	Sensitivity	0]* Sensitivity per Linewidth	Detection Range
	(nm/RIU)		(Refractive Index)
[42]	252	83	1.33–1.38
[43]	260.8	33	1.333–1.373
[44]	199	19	1.336–1.371
[45]	286.2	14	1.33–1.39
[46]	428.1	-	1.317–1.445
[47]	510	320	1.59–1.61
[48]	596	7.5	1.00–1.05
[49]	500	4.5	1.00–1.30
[50]	8028	4.0	1.00–1.20
This work	566.6	35.4	0.77–1.41
